# Quality Improvement of Venous Thromboembolism Prophylaxis in Neurological Surgery: Ochsner Health System Protocol

**DOI:** 10.2196/57278

**Published:** 2025-10-14

**Authors:** Velina S Chavarro, Jayanth Mosalakanti, Stephen Z Shapiro, Yana Bukovskaya, Stephanie Youssef, Steven Deitelzweig, Joseph Keen, Cuong Bui, Lora Kahn

**Affiliations:** 1 Department of Neurosurgery Ochsner Health System Jefferson, LA United States; 2 Department of Neurosurgery Tulane University School of Medicine New Orleans, LA United States; 3 Neurocritical Care Ochsner Health System Jefferson, LA United States; 4 Clinical Pharmacy - Cardiology Ochsner Health System Jefferson, LA United States; 5 Department of Hospital Medicine Ochsner Health System Jefferson, LA United States

**Keywords:** chemoprophylaxis, incidence, neurological surgery, neurosurgery, neurosurgery society guidelines, Ochsner Health System, patient-centered, patient-centered care, postoperative care, postoperative complication, quality improvement, venous thromboembolism, venous thromboembolism prophylaxis, VTE

## Abstract

**Background:**

Venous thromboembolism (VTE) is a common postoperative complication. The perioperative incidence of VTE in patients undergoing neurosurgery on VTE chemoprophylaxis is consistently between 1.7% and 3.5%, excluding patients with trauma. While the majority of VTEs are asymptomatic, symptomatic VTEs may result in serious adverse events or death. There are limited neurosurgery society guidelines making VTE prophylaxis recommendations for patients undergoing cranial or spine neurosurgery. Several societies have published VTE prophylaxis guidelines for neurosurgery, but inconsistent recommendations have led to varied practices based on neurosurgeon preference.

**Objective:**

This study aims to assess whether implementing a single protocol of VTE guidelines at a large hospital system department of neurosurgery would decrease the rate of VTE incidence by 50% within a 12-month period.

**Methods:**

This project uses a department-wide protocol of chemical prophylaxis for admitted patients after neurological surgery. This quality improvement (QI) project has been designed and reported in accordance with the SQUIRE 2.0 (Standards for Quality Improvement Reporting Excellence) guidelines. SQUIRE is the most appropriate framework for reporting system-level interventions aimed at improving health care quality, safety, and outcomes. This manuscript addresses all relevant SQUIRE elements. The primary outcome is the VTE incidence over 12 months following protocol implementation. Secondary outcomes include the incidence of pulmonary embolism, length of hospital stay, and unexpected treatment of patients, such as therapeutic anticoagulation, thrombolysis, or emergency surgical or catheter-directed embolectomy. Balancing measures and process measures are also considered. Interim analyses with department reports will be performed every 3 months, but every VTE event will trigger an analysis of the variables described in this protocol. Analyses will consider barriers to change and adequacy of the protocol to guide protocol modifications and continuous implementation of improvements.

**Results:**

This QI project was approved on February 16, 2023 (protocol ID NEU20230216-01). Adaptation and training began on August 1, 2023. As of May 2024, data collection is ongoing and scheduled to conclude by December 2024. Final data analysis is anticipated in March 2025, with results submitted for publication by the end of 2025.

**Conclusions:**

It is anticipated that this QI project implementing a department-wide protocol for chemical prophylaxis in patients undergoing neurosurgery will improve the rates of VTE in patients on our service. In addition to a discussion of our results, we provide a comprehensive review of the literature and establish a gap in consistent published guidelines for VTE prophylaxis in patients undergoing neurosurgery. Results from this QI project will provide evidence for the effectiveness of a unified protocol of VTE prophylaxis in this patient group. Furthermore, outcomes from this study may be used as the basis for guideline recommendations and will benefit not only our department but may be used for QI of patient care at other hospitals.

**International Registered Report Identifier (IRRID):**

DERR1-10.2196/57278

## Introduction

### Overview

Venous thromboembolism (VTE) is a common postoperative complication. The incidence of VTE in perioperative patients undergoing neurosurgery is consistently between 1.7% and 3.5%, even among patients on pharmacologic prophylaxis, chemoprophylaxis (cPpx), excluding trauma patients [1,2]. A 2015 American College of Surgery (ACS) National Surgical Quality Improvement Program (NSQIP) retrospective analysis [3] of 10,477 patients who underwent cranial surgery across the United States reported an incidence of VTE of 3%-3.4%. Similarly, a single hospital system (Mayo Clinic) analysis of 1622 craniotomies for tumor resections between 2012 and 2017 documented a 3% incidence of VTE [4]. A large retrospective hospital system study (at the University of California) reported the incidence of VTE in patients who underwent spine surgery for nonmalignant versus malignant pathology as 0.5% versus 2.0%, respectively [5]. Incidence of VTE among patients with trauma is, however, much higher. In patients with neurosurgical trauma, the American Association for the Surgery of Trauma (AAST, 2021) cites 20%-30% VTE incidence for traumatic brain injury (TBI) [6,7], while the ACS Best Practices (2022) cites 40%-70% incidence for patients with spinal cord injury (SCI) [8]. As per the North American Spine Society’s (NASS) most recent guidelines on antithrombotic therapies in spine surgery, there exists no high-quality study to establish definitively the incidence of VTE in nonelective spine surgery [5,9]. Most published studies assessing VTE incidence are retrospective analyses of NSQIP or other large hospital system data.

In addition, currently there are no published neurosurgery society guidelines making VTE prophylaxis recommendations for patients undergoing cranial or spine neurological surgery, including the Congress of Neurological Surgeons (CNS), American Association of Neurological Surgeons (AANS), or NASS. Multiple non-neurosurgical societies have commented and made a variety of recommendations for the prophylaxis of VTE in patients undergoing neurosurgical procedures. The American Society of Hematology 2019 Guidelines suggest against routine cPpx in patients undergoing neurosurgery in general but suggest all patients should receive mechanical prophylaxis (mPpx) [10]. They highlight that cPpx should be considered in patients at the highest risk, such as patients experiencing prolonged immobility or those undergoing a major neurosurgical procedure and suggest low molecular weight heparin (LMWH) over low-dose unfractionated heparin (UFH). Similarly, the American College of Chest Physicians (ACCP) 9th Edition Guidelines 2012 suggest mPpx with sequential compression device (SCD) over UFH, LMWH, or no prophylaxis for patients undergoing cranial or spine surgery, and cPpx for patients with very high risk of VTE (ie, malignant disease) once hemostasis is obtained and risk of bleeding decreases [11,12]. The ACCP guidelines recommend against an inferior vena cava (IVC) filter use for primary VTE prevention [13]. The new ACCP 2022 revised guidelines do not mention specific anticoagulation for patients undergoing neurosurgical procedures [12]. For patients with central nervous system trauma, including TBI, SCI, and so on, the AAST VTE Ppx in the intensive care unit (ICU) 2021 Guidelines suggest prophylaxis within 24-72 hours of admission pending stability head computed tomography based on Berne-Norwood criteria [14] with either Enoxaparin 30 mg every 12 hours (with Xa target of 0.2-0.4 mg/mL) or UFH 5000 U every 8 hours [7] (Multimedia Appendix 1).

Moreover, most guidelines do not comment on patients with a BMI>30 kg/m^2^ and do not include this patient population as a confounder in analyses. Based on our summary of the literature, the most frequently used and recommended methods of VTE prophylaxis include the following:

1. mPpx with sequential compressive devices, which are demonstrated to reduce the risk of deep vein thrombosis (DVT) compared with control treatment (odds ratio [OR] 0.85, 95% CI 0.5-1.50); however, this is based on low-quality evidence [15]. According to a recent Cochrane review, mPpx reduces the incidence of DVT (relative rate [RR] 0.43, 95% CI 0.25-0.73), but cPpx is more effective than mPpx at reducing the incidence of DVT (RR 0.48, 95% CI 0.25-0.95) [16].
2. Chemical prophylaxis with LMWH is considered to reduce the risk of DVT compared to UFH (RR 0.68, 95% CI 0.50-0.94) [15,17]. Patients who received both mPpx and cPpx with LMWH had a lower risk of DVT [16,18]. UFH alone has shown a strong reduction in VTE incidence compared to placebo (RR 0.27, 95% CI 0.10-0.73) [19,20].
3. IVC filter is an alternative prophylaxis for patients at high risk of bleeding in whom cPpx has either been shown ineffective or is contraindicated. Patients receiving an IVC filter had lower incidence of subsequent pulmonary embolism (PE; OR 0.5, 95% CI 1.17-2.48), nonsignificant lower PE–related mortality (OR 0.51, 95% CI 0.25-1.05), and no change in all-cause mortality (OR 0.91, 95% CI 0.70-1.19) [21].

While the majority of VTEs are asymptomatic, symptomatic VTEs may result in serious adverse events or death [4]. Therefore, prophylaxis of VTE plays a major role in reducing adverse events and unexpected mortality in patients undergoing neurosurgery. An ACS NSQIP data query for Ochsner Department of Neurosurgery performed on August 1, 2022, for the period of July 1, 2020, to June 30, 2021, yielded incidence rates of VTE as follows: a total of 8 events per 320 total neurosurgery cases for a rate of 2.5%, whereas the predicted rate of VTE was calculated to be 2.83% and the expected rate based on the national average rate of VTE was calculated to be 3.27%. This has resulted in an OR calculation of 0.86 (95% CI 0.52-1.42). When stratified by the type of neurosurgery, the VTE incidence rate was 0.76%, where a VTE event occurred in 1 case out of a total of 132 spine neurosurgery cases. The predicted rate for spine neurosurgery was 1.78% and the expected rate for spine neurosurgery cases was 2.24%, with an OR of 0.77 (95% CI 0.37-1.61). The rate of VTE after neurosurgery for brain tumors at Ochsner was 3.52%, based on 5 cases out of 142 total brain tumor neurosurgery cases. The predicted rate of VTE incidence for patients undergoing neurosurgery for brain tumor was 3.45% and its expected rate was 3.42%, with an OR of VTE incidence among patients after brain tumor neurosurgery of 1.01 (95% CI 0.63-1.63).

Although the incidence of VTE at Ochsner Health Department of Neurosurgery is within the expected range of nationwide data [1-3], the lack of a department- or hospital-based protocol for postoperative VTE prophylaxis results in a heterogeneity of patient care and may contribute to unexpected outcomes. Current practice dictates that individual physicians apply their chosen set of VTE prophylaxis guidelines [17]. This further contributes to a heterogeneity of patient care since most guidelines provide inconsistent recommendations (Multimedia Appendix 1). This creates a gap in patient care that could potentially be bridged through a consistent set of department VTE prevention guidelines, which would aim to diminish patient care variability and reduce the incidence of VTE below the national average rates.

### Objective

A department-wide protocol will aim to decrease the rate of VTE by providing a set of consistent guidelines for physicians, physicians-in-training, advanced practice providers, and other clinical staff involved in direct patient care. The objective of this quality improvement (QI) project is to assess the effect of implementing a practice-wide protocol of VTE prophylaxis on the incidence of VTE at Ochsner Health among admitted patients undergoing nonemergent neurosurgery procedures. Implementing a department-wide VTE prophylaxis protocol based on individualized patient risk aims to ensure patient-centered care and enhance the safety and effectiveness of postoperative care. Moreover, we propose that establishing consistent guidelines for VTE prophylaxis in postoperative patients will contribute to efficiency and minimize errors based on heterogeneity of care. A further step to improve patient-centered care is the consideration of both patients’ BMI and weight when determining cPpx dosing–a rarely applied approach that is particularly germane for our patient population in the southern United States.

## Methods

### Overview

This project is well aligned with the Institute of Medicine’s 6 domains of quality health care and was designed according to the Institute for Healthcare Improvement steps to a good QI project [22]. It aims to avoid harm to patients while being effective in providing prophylaxis according to a risk-based approach. Specifically, the adaptive design assessing outcomes and process measures and implementing incremental change on a quarterly basis ensure the safety of this QI project. Our protocol follows the Agency for Healthcare Research 2017 guidelines to ensure patient safety while implementing this protocol [23]. This quality improvement project has been designed and reported in accordance with the SQUIRE 2.0 (Standards for Quality Improvement Reporting Excellence) guidelines. SQUIRE is the most appropriate framework for reporting system-level interventions aimed at improving health care quality, safety, and outcomes. This manuscript addresses all relevant SQUIRE elements [24]. Further, this project is patient-centered and aims to ensure equitable care for patients by consistently including all eligible individuals in the protocol without discriminating based on demographics. Finally, it is a timely and efficient protocol, reducing inconsistent application of variable guidelines and thus potentially avoiding supplies and staff waste. The assessment of the impact of this protocol on VTE incidence is at 12 and 24 months post implementation, with interim analyses every 3 months to ensure continuous quality improvement. The goal is a 50% decrease in the overall VTE incidence in patients undergoing neurosurgery from 2.5% to 1.25%, with a greater decrease in incidence of VTE among patients undergoing neurosurgical procedures for malignant pathology (cranial or spine). Targeting a reduction to 1.25% represents an achievable improvement that aligns with outcomes seen in successful QI initiatives with standardized protocols [25-27]. For example, the Mercy Hospital Springfield Neurosurgery VTE quality improvement initiative found that VTE occurrence rates in neurosurgery decreased from 3.97% at the time of protocol initiation to 0.72% in January 2020 [26]. All patients admitted to the neurosurgery service for postoperative care will be managed according to the new protocol after the implementation date. The incidence of VTE among patients admitted during the previous 12 months will serve as a control.

### Ethical Considerations

This project did not meet the threshold for institutional review board (IRB) review and approval per Ochsner Health Systems (IRB) guidelines. Human participants were not consented for individual participation in this project. Patient data were stored behind a Health Insurance Portability and Accountability Act (HIPAA)–compliant hospital-wide internet firewall. All patient data are deidentified and anonymized before analysis and dissemination. Participants were not compensated for providing their data or for participation in this project. All staff involved in this protocol adhere to HIPAA. This QI initiative is purely a retrospective study of chart data or similar, and consent was not required for the procedures performed [23,27-31]. As such, it falls under the definition of nonhuman participants research according to both the US Department of Health and Human Services (HHS) Office for Human Research Protections (OHRP) and our institutional IRB. This project was reviewed by the Ochsner Health Institutional Review Board and was deemed exempt from IRB approval based on institutional guidelines for QI activities. The key criteria met include the following 4 criteria:

1. The intervention (VTE prophylaxis protocol) was developed and implemented solely for the purpose of improving care within Ochsner.

2. The protocol will be implemented at the department level as a clinical standardization initiative, with all patients receiving care under the new protocol as part of routine care.

3. No patient-identifiable data are used in any dissemination of findings. All data are retrospective, deidentified, and securely stored in a HIPAA-compliant system.

4. There are no additional risks or burdens that were imposed on patients outside of standard care practices. A formal determination of exemption was made under internal protocol ID NEU20230216-01, approved by our department leadership on February 16, 2023.

Ethics approval was not required for this protocol per local IRB requirements. However, all authors strictly adhere to patient privacy and confidentiality according to the US HIPAA.

### Outcomes

The primary outcome measure is the rate of VTE. Benchmark data for the primary outcome is derived from ACS NSQIP VTE rates for Ochsner Neurosurgery and the nationwide ACS NSQIP VTE rate. Benchmarks are 2.5% for Ochsner Neurosurgery and 3.27% for the national incidence rate [3]. Secondary outcome measures include the rate of PE, the rate of symptomatic DVT, and the rate of PE-associated mortality.

Process measures have an impact on the primary and secondary outcomes and will include staffing at the time of VTE diagnosis, volume of patients per resident physician, and the percentage of patients at high risk. Balancing measures include the complexity of patients based on VTE risk and history of VTE despite prophylaxis, medication reconciliations, communication with other services, and volume of patients on the neurological ICU. Procedure and patient risk factors of VTE will be included in the analysis as confounding variables. Unintended consequences such as supratherapeutic anticoagulation or bleeding events, delays in mobilization or surgery due to protocol requirements, or variation in adherence will be monitored as well. The mentioned unintended consequences will be monitored through case review and clinical incident reports, and adjustments to the protocol will be made through the Plan-Do-Study-Act cycle framework.

### Selecting Change

**Figure 1:**
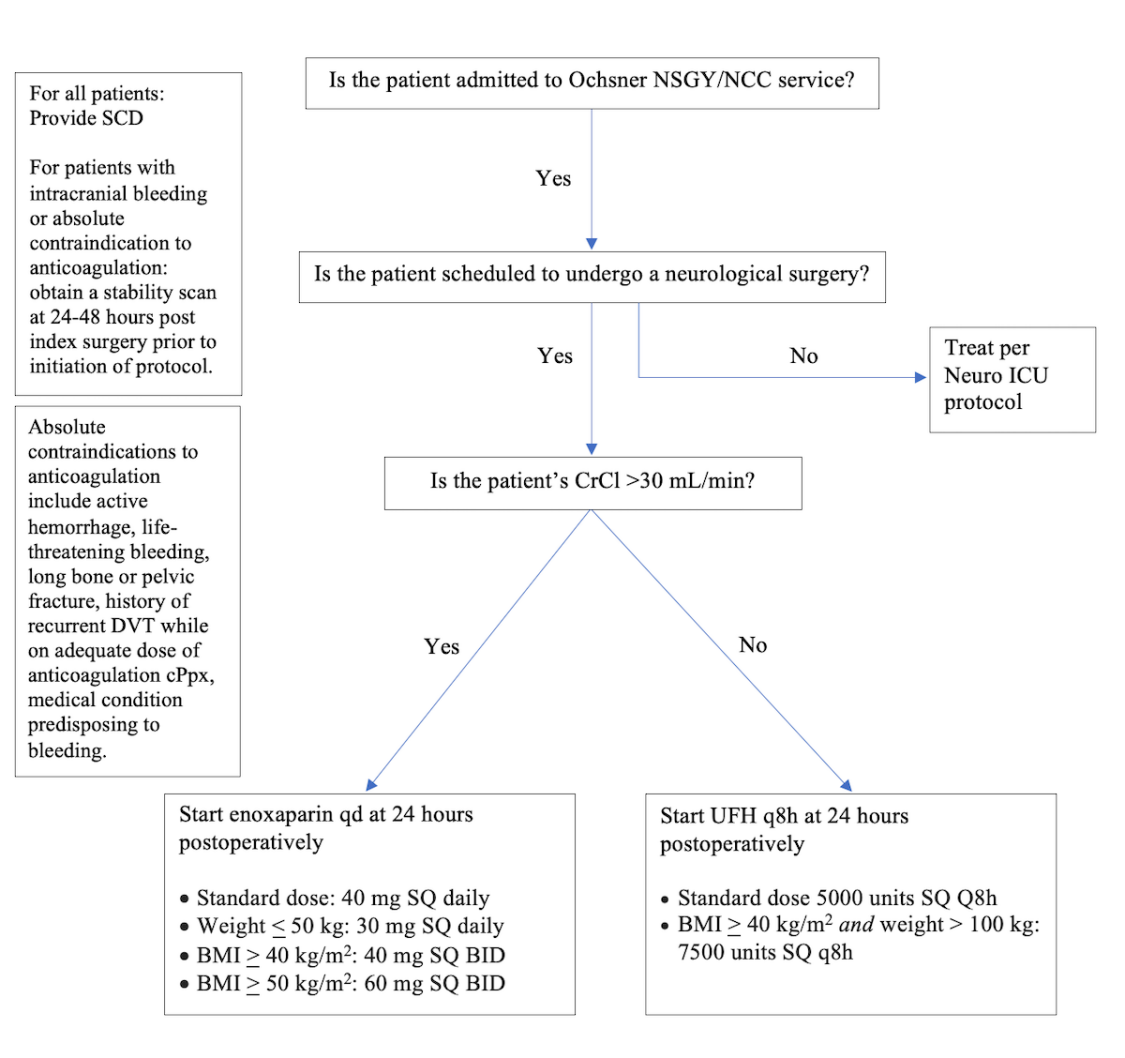
Protocol flowchart. BID: twice daily; CrCl: creatinine clearance; DVT: deep vein thrombosis; ICU: intensive care unit; NCC: Neuro Critical Care; NSGY: neurosurgery; Q8h: every 8 hours; qd: daily; SCD: sequential compression device; SQ: subcutaneously.

Two major changes will be implemented. Implementation of a risk-based protocol for VTE prophylaxis for all Department of Neurosurgery patients undergoing elective surgery, and training of direct patient care staff on the protocol across the Departments of Neurosurgery and Neuro Critical Care (NCC). The following steps outline the QI protocol:

1. For all patients admitted to the Neurosurgery or NCC service and undergoing a neurological surgery, use mPpx with SCD intraoperatively and until discharge. An exception is patients in whom mPpx with SCD is contraindicated, such as patients with known DVT or lower extremity injury.
2. For patients with intracranial hemorrhage, we will obtain stability imaging at 24-48 hours before starting cPpx.
3. For all patients admitted to the Neurosurgery or NCC service and undergoing an elective neurological surgery, provide cPpx starting at 24 hours until discharge, unless an absolute contraindication to anticoagulation is present (per #5).
4. Check creatinine clearance (CrCl) and choose cPpx with either enoxaparin if CrCl is >30 mL/minutes or UFH if CrCl≤30 mL/minutes. Dosing for all patients is according to weight and BMI, as obesity is an independent risk factor for hypercoagulability. Small studies have shown an advantage of weight-based dosing in achieving target anti-Xa levels with enoxaparin administration [32,33]. A clinical trial has also demonstrated a reduction in VTE rates for patients on UFH cPpx when using both BMI and weight (weight>100 kg and BMI≥40 kg/m^2^) [20]. For weight-based enoxaparin prophylaxis, the standard dose is 40 mg subcutaneously daily. For patients weighing less than 50 kg, the dose is 30 mg subcutaneously daily. Patients with a BMI more than 40 kg/m² should receive 40 mg subcutaneously twice daily, and those with a BMI more than 50 kg/m² should receive 60 mg subcutaneously twice daily. For weight-based unfractionated heparin prophylaxis, the standard dose is 5000 units subcutaneously every 8 hours. Patients with a BMI more than 40 kg/m² and a weight over 100 kg should receive 7500 units subcutaneously every 8 hours.
5. Per AAST VTE prophylaxis in the ICU (2021) [7], an absolute contraindication to cPpx for patients hospitalized with trauma is active hemorrhage or recent spine or intracranial surgery. This is in addition to absolute contraindications to therapeutic anticoagulation, including a prior complication while on anticoagulation therapy or recurrent VTE while on an adequate dose of cPpx, or ongoing risk due to life-threatening bleeding. Risk of life-threatening bleeding includes long-bone fractures, severe pelvic fractures, plus a long-bone fracture, TBI, SCI, and medical conditions predisposing to bleeding. Patients who meet these criteria will be assessed with a stability scan before inclusion in this protocol. Relative contraindications to cPpx include epidural hematoma, risk of wound complications, and neurologic decline. Patients with relative contraindications will be included in the protocol.
6. VTE therapy will be continued until the patient is discharged from the hospital and no longer immobilized.
7. Training on this protocol was provided to all health care providers involved in postoperative neurosurgery patient care. A flowchart is displayed in common work areas (Figure 1). This training took place between Inservice in September and October 2023.

Continuous improvement is built with small incremental changes, using a systematic scientific approach to test their impact and feasibility. The Plan-Do-Study-Act cycle will be used as a model on which to structure tests of change. This includes continuous analysis of errors and successes of the protocol and subsequent protocol adaptation for the duration of the project. Dynamic data will be assessed through a run chart with data points added monthly, but with interim analysis every 3 months. Interim analyses will be discussed before changing the protocol for such quality improvement adaptations. A formal statistical analysis will be performed with a *t* test comparing NSQIP and postprotocol end-of-study VTE rates at both 12 and 24 months post implementation. Stratification based on malignancy and cranial versus spine procedures will also be assessed, as will be the confounding process and balancing measures.

Missing data will be handled through checking at each interim review. Any record missing critical outcome or key covariate information will be excluded from regression analyses. In addition, we will conduct sensitivity analyses to assess the impact of any missing data on VTE rates. Discrepancies will be resolved by consensus and reported, if indicated. Interim analyses with department reports will be performed every 3 months, but every VTE event will trigger an analysis of the variables described in this protocol. Analyses will consider barriers to change and adequacy of the protocol to guide protocol modifications and continuous implementation of improvements.

Additional guidance has been incorporated to inform future iterations of the protocol, including considerations for patients with comorbid atrial fibrillation or those already on antiplatelet therapy [34]. Implemented changes will be made based on recent evidence in the literature [35].

## Results

The desired timeframe for implementing the Neurosurgery VTE prophylaxis protocol is within 6 months of QI project approval, with 1 month of “adaptation time.” Adaptation time is defined as the time between training the staff involved in the project and starting the timeline for outcomes assessment. Department project approval occurred on February 16, 2023, and the project adaptation time began on August 1, 2023.

Data collection is scheduled for completion in 2024, with analysis expected by March 2025. We anticipate a 50% reduction in VTE incidence (from 2.5% to 1.25%) following implementation of a standardized department-wide cPpx protocol. The primary outcome is the incidence of VTE. To assess whether implementation of the protocol results in a meaningful improvement compared to the standard of care, we will use a pre-post design comparing outcomes in the 12 months before and 12 months after the protocol implementation. We will evaluate differences in VTE rates using chi-square or Fisher exact test to compare the proportion of VTE events pre-implementation versus post-implementation. The justification for the chi-square test is that it is appropriate for large samples with expected counts ≥5. The Fisher exact test will be used as a backup for smaller subgroup comparisons where expected frequencies are low. In order to mitigate confounding, we will use multivariable logistic regression, using VTE occurrence as the dependent variable, with covariates including patient-level and procedural risk factors such as BMI, malignancy, surgery type (cranial vs spine), and immobility. The justification is that regression modeling will help isolate any independent effects of the protocol on VTE rates. To account for continuous secondary outcomes, we will perform stratified subgroup analyses for patients with malignancy, those undergoing cranial versus spine procedures, and those receiving LMWH versus UFH. The justification for this is that we will protect against or identify subgroups that experience differential VTE risk. A stratified analysis will determine if the protocol is equally effective across populations.

Outcomes of this QI project will be reported to the Ochsner Health Department of Neurosurgery as well as hospital administration. Results will also be shared with the scientific and health care quality improvement community. Consideration of “lessons learned” and suggestions for implementing change in order to improve patient outcomes will be made. In addition, consideration of barriers to change and recommendations for improving patient care will be discussed. This project will provide an evidence base for the implementation of the proposed protocol, and its success or failure to achieve the protocol's aims will be equally important for future hospital patient care quality improvement efforts. Considering there are limited neurosurgical society guidelines on VTE prophylaxis in perioperative patients, results from this project can be used to assess the efficacy of our specific protocol in guiding future guidelines specific for patients undergoing neurosurgery. The overarching objective of this quality improvement initiative is to advance the evidence of the effectiveness of anticoagulation prophylaxis in preventing perioperative VTE morbidity and mortality. As previously identified, the determination of whether implementation of this protocol was a success compared to the current standard-of-care success criteria will be measured against a ≥50% reduction in VTE incidence compared to baseline (from 2.5% to ≤1.25%). Secondary success measures include reduction in symptomatic PE and DVT events, and improvement in protocol adherence across clinical teams.

## Discussion

### Expected Findings

Our review of the literature (summarized in Multimedia Appendix 1) demonstrated that all published guidelines by non-neurosurgical societies are largely inconsistent. While some suggest against the use of pharmacological prophylaxis in the majority of patients undergoing neurosurgery, others suggest stratifying patients based on risk of VTE, including those with malignancy or immobilization, and providing pharmacological prophylaxis in the high-risk group. Moreover, the choice of anticoagulant is different between published guidelines, and most do not consider weight-based dosing.

In devising our protocol, we borrowed heavily from the trauma and neurocritical care literature when choosing the timing, agents, and dosing for VTE cPpx. NASS working group on VTE prophylaxis [9] notes that the safety and utility of cPpx is controversial, and the literature does not support a specific perioperative start time point nor duration of cPpx. However, the working group acknowledges that LMWH initiated on the day of surgery may be safe for patients undergoing elective spine surgery and makes a consensus-based recommendation for it in their 2009 guidelines [9]. On the other hand, the ACCP recommends initiating cPpx in trauma neurosurgical patients within 24-72 hours of admission pending a stability scan [7]. The NCC guidelines [36] alternatively note that VTE cPpx should be initiated after 24 hours of admission for patients with aneurysmal subarachnoid hemorrhage, after 48 hours for patients with intracerebral hemorrhage, and within 24-72 hours for patients with TBI, but not before. We have elected to initiate cPpx for all admitted patients on the neurosurgery service within 24-48 hours of scheduled surgery. Patients who have a contraindication to anticoagulation or are at a high risk of intracranial bleeding will enter the protocol pending a stable head computed tomography.

In terms of the choice of agent for VTE cPpx, Nyquist et al [36] recommend prophylaxis with UFH for patients after a nontraumatic subarachnoid hemorrhage, and either UFH or LMWH in other patients with intracerebral hemorrhage or patients with neurosurgical trauma. The AAST [7], on the other hand, notes that even though LMWH and UFH have comparable bleeding complications, LMWH results in a lower incidence of PE in trauma neurosurgical patients. Consistent with this, we selected LMWH for our protocol as the primary anticoagulant agent. However, patients with poor renal function, assessed as CrCl<30 mL/minutes, will receive UFH instead.

Another critical aspect of our protocol is the introduction of weight-based dosing. Samuel et al [20] have shown that using 7500 Units of UFH in patients on the neurocritical care service who weigh >100 kg does not lead to increased complications. Similarly, Sebaaly and Covert [33] summarize the literature on dose adjustments of LMWH to 30 mg every 8 hours for patients weighing <50 kg or having a BMI of <18 years, reporting comparable complications between higher doses and lower doses of LMWH. The AAST also recommends weight-based dosing for LMWH. Although our study design does not randomize patients, it will allow us to demonstrate the practical challenges of implementing a department-wide VTE prophylaxis protocol in reducing VTE incidence and associated morbidity and mortality.

It is further important to emphasize that VTE cPpx is often used in addition to and not instead of mPpx. Although there is evidence suggesting cPpx may be more effective in reducing the risk of VTE [15,16], all of our patients receive mPpx during their admission unless contraindicated. Consequently, our QI project is not intended to assess the significance of mPpx but rather to assess whether a standardized cPpx protocol may contribute to reducing the overall incidence of VTE and DVT in our patient population.

It is important to consider that a subset of patients may already be on a therapeutic dose of anticoagulation or antiplatelet medications for a history of DVT or PE before hospitalization. Such medications are typically held the evening before a neurosurgical procedure, or in cases of emergency surgery, may be reversed on an individual patient basis. Postoperatively therapeutic dose of anticoagulation or antiplatelet medication may be restarted in these patients. Such patients also continue their regular dose upon discharge. We do not provide a prescription for mechanical VTE prophylaxis other than early mobilization with physical therapy.

### Limitations

As this is a nonrandomized pre-post QI design, the project is susceptible to selection bias and performance bias. Examples of such biases include changes in staffing patterns or seasonal admission trends, which may influence VTE rates. We acknowledge this limitation and address it through stratified subgroup analyses and logistic regression to adjust for known confounders. In addition, although we use multivariable regression to control for common risk factors (BMI, malignancy, immobility, and surgery type), unmeasured, or residual confounding may still influence outcomes. We recognize this as a limitation in attributing causality to the intervention. Finally, there is potential for missing or incomplete documentation in retrospective chart review. We aim to address this through predefined rules for data exclusion and sensitivity analysis, as described in the Methods section.

## Appendix

Multimedia Appendix 1 Guidelines summary.

## Abbreviations

AANS: American Association of Neurological Surgeons
AAST: American Association for the Surgery of Trauma
ACCP: American College of Chest Physicians
ACS: American College of Surgery
CNS: Congress of Neurological Surgeons
cPpx: pharmacologic prophylaxis, chemoprophylaxis
CrCl: creatinine clearance
DVT: deep vein thrombosis
HHS: US Department of Health and Human Services
HIPAA: Health Insurance Portability and Accountability Act
ICU: intensive care unit
IVC: inferior vena cava
IRB: institutional review board
LMWH: low molecular weight heparin
mPpx: mechanical prophylaxis
NASS: North American Spine Society
NCC: Neuro Critical Care
NSQIP: National Surgical Quality Improvement Program
OHRP: Office for Human Research Protections
OR: odds ratio
PE: pulmonary embolism
QI: quality improvement
RR: relative rate
SCD: sequential compression device
SCI: spinal cord injury
SQUIRE: Standards for Quality Improvement Reporting Excellence
TBI: traumatic brain injury
UFH: unfractionated heparin
VTE: venous thromboembolism
