# Supplementary material for: Quality Improvement of Venous Thromboembolism Prophylaxis in Neurological Surgery: Ochsner Health System Protocol
Source: JMIR Res Protoc. 2025 Oct 14;14:e57278. doi: 10.2196/57278 (PMC12569481; doi:10.2196/57278)
Supplement: Multimedia Appendix 1 [file resprot_v14i1e57278_app1.docx]

APPENDIX A. Guidelines and Cofounders Summary.

ACS has guidelines on managing perioperative AT medication for patients on chronic anticoagulation including CAD, PAD which may be very applicable to our patient population.^24^

Most guidelines do not comment on patients with BMI>30 and do not include this patient population as a confounder in analyses.^6^

1. Guidelines for VTE Ppx in Surgical Patients
2. American Society of Hematology (ASH) 2019 guidelines for prevention of VTE in surgical hospitalized patients^10^

In the absence of lower extremity injuries, all patients major trauma should receive mechanical prophylaxis.

1. American College of Chest Physicians (ACCP) Guidelines 9^th^ Edition (2012) and Second Update (2021). With 2^nd^ Update in 2021.^25^
   1. Prevention of VTE in Non-Orthopedic Surgical Patients (2012)^11^
   2. Prevention of VTE in Orthopedic Surgery Patients (2012)^26^

Hip fracture surgery is evaluated by ACCP to be highest risk category for VTE and all hip fractures should receive VTE ppx, cPpx or mechanical^27^

1. American College of Surgeons (ACS) Guidelines on Perioperative Management of Anti-thrombotic Medications may be generally applicable to our patient population as it provides guidelines on perioperative management of patients who are on chronic anticoagulation.^24^
2. American Association for the Surgery of Trauma (AAST) Critical Care Committee Clinical Consensus VTE Ppx in the ICU 2021^6^
   1. Rec. 8.4.1. For major trauma patients ACCP suggests LDUH, LMWH or mechanical ppx over no ppx

B. Guidelines for VTE Ppx in Neurosurgical Patients

Cranial. Currently there are no Neurosurgery Society guidelines for VTE prophylaxis in cranial neurosurgery patients.

1. ASH 2019 Guidelines^10^ for Prevention of VTE in Surgical Hospitalized Patients has made two conditional recommendations based on very low evidence of effect for Neurological surgery (Rec. 19 and 20)

a. ASH 2019 Guidelines panel suggests against pharmacological prophylaxis. All patients should receive mechanical prophylaxis. In patients at higher risk, including prolonged immobilization, pharmacological prophylaxis may be warranted.

b. For patients undergoing neurosurgical procedure for whom pharmacological prophylaxis is used ASH suggests LMWH over LDUH.

Overall, mPpx is recommended, while cPpx is considered for high-risk patients.

1. ACCP 9^th^ Edition Guidelines 2012^11^: Prevention of VTE in Non-Orthopedic Surgical Patients have Recommendations for the following surgery categories: cranial surgery (Section 6), spine surgery (Section 7) and trauma, including brain and spine trauma (Section 8).
2. Rec 6.4.1. For craniotomy patients ACCP suggests mechanical prophylaxis preferably with IPC over no prophylaxis or pharmacologic prophylaxis
3. Rec 6.4.2. For craniotomy patients at very high risk for VTE (malignant disease) ACCP suggests pharmacologic prophylaxis over mechanical prophylaxis once hemostasis is obtained and risk of bleeding decreases.
4. Rec. 8.4.1. For major trauma patients at high risk for VTE including acute SCI, TBI and spinal trauma surgery, ACCP suggests adding mechanical prophylaxis to pharmacologic prophylaxis.
5. Rec. 8.4.4. For major trauma patients, IVC filter should not be used for primary VTE prevention
6. Rec. 8.4.5. For major trauma patients, periodic surveillance with VCU should not be performed.

1. AAST VTE Ppx in the ICU 2021^6^ is almost entirely dedicated to neuro trauma VTE prophylaxis including core questions of interest in this review.
   1. Thromboprophylaxis should be initiated as soon as possible following TBI and within 24-72 hours of admission, pending stability scans.

Berne-Norwood criteria suggest that in the absence of multiple contusions, isolated SAH, IVH, SDH/EDH <8mm ppx initiated at 24 hours post injury pending stability head CT

- 1. LDUH or LMWH may be used for VTE ppx in TBI, although LMWH may be superior.

Per Byrne et al. TQIP propensity matching LMWH had lower rates of PE vs UFH (1.4% vs 2.4%).

- 1. In TBI, both UFH 5000U q8h and LMWH enoxaparin 30mg q12h are most commonly used, assess anti-factor Xa levels within targets of 0.2-0.4mg/ml to ensure minimal risk of ICH progression. If levels are below target, increase Lovenox by 10mg and recheck levels q4h

1. ACS TBI Best Practices 2022^5^
   1. Patients with TBI are at a high risk of VTE (20-30%) even with appropriate mechanical prophylaxis. Mechanical prophylaxis should be initiated in all TBI patients, and cPpx considered within the first 72 hours following TBI in most patients. Earlier pharmacologic ppx is safe in patients with low risk of progression of intracranial bleed. Delay in cPpx >4 days is associated with increased risk of VTE.

Spine. The only neurosurgery society guidelines are applicable to spine surgery patients and are the NASS 2009 Guidelines^9^.

1. NASS 2009 Guidelines^9^ are currently being updated, protocol of systematic review for current evidence was published in 2021^8^. Although the group concluded there was no high-quality evidence to determine the incidence of VTE, DVT and PE in prophylaxed or unprophylaxed patients undergoing spine surgery, they recommend:
   1. III.A.4. Clinical examination alone is not a reliable method to confirm the dx of DVT, venography or doppler US should be used to confirm suspected DVT in post-op spine patients.
   2. IV.A. Mechanical compression devices in LE are suggested in elective spine surgery to decrease the risk of VTE.
   3. IV.B. Mechanical compression device should be used prior to or at beginning of spine surgery until the patient is fully ambulatory.
   4. IV.C. Chemoprophylaxis in spine surgery patients is controversial as elective common procedures carry a very low risk of VTE and do not warrant cPpx.

Initiating LMWH preoperatively can decrease the incidence of VTE, but increases bleeding complications. LMWH can be safety started the day of elective spine surgery, but the working group recommends caution in use of LMWH in elective spine surgery unless other risk factors are present.

There is no recommendation based on current literature for duration of cPpx, but the group recommends this is individualized and based on patient comorbidities including heart valve, previous DVT, stent ppx and ambulatory/neuro patient status.

1. ACS Spinal Cord Injury Best Practices 2022^7^

ACS SCI 2022 suggests initiation of cPpx as early as medically possible, typically within 72 hours to reduce risk of VTE. Spinal cord injury patients have an elevated risk of VTE of 40-70%.

Multiple studies show that in patients w traumatic SCI requiring surgical intervention, there was no significant increase in post op complications when LMWH was initiated in the acute period after surgery (24-72h). Full anticoagulation is associated with increased risk of bleeding and no benefit over ppx AC.

1. ACCP 2012^6^
   1. 7.4.1 For spinal surgery, suggest mPpx with IPC over no ppx, UFH or LMWH
   2. 7.4.2. For spinal surgery of patients at high risk for VTE such as malignancy or anterior-posterior approach, suggest adding cPpx to mPpx once hemostasis is established and risk of bleeding decreases
   3. For major trauma including spinal trauma surgery suggest adding mPpx to cPPx

C. Specific Procedure and Risk Factors Included as Cofounders in the Analysis

The following procedure- and patient-level risk factors will be included as confounding variables in the analysis:

Procedure-related variables:
- Cranial vs spine surgery
- Elective vs emergent procedure
- Presence of malignancy
- Intraoperative blood loss
- Duration of surgery
- Use of intraoperative anticoagulants
- Admission to neurocritical care unit

Patient-related risk factors:
- BMI and weight class (e.g., BMI >40 kg/m²)
- Age
- History of prior VTE
- Pre-existing atrial fibrillation or antithrombotic use
- Renal function (e.g., CrCl <30 mL/min)
- Mobility/immobility status
- Smoking status
- Comorbidities (e.g., cancer, diabetes, coagulopathy)
- Length of hospital stay
